# Melatonin and its metabolites protect human melanocytes against UVB-induced damage: Involvement of NRF2-mediated pathways

**DOI:** 10.1038/s41598-017-01305-2

**Published:** 2017-04-28

**Authors:** Zorica Janjetovic, Stuart G. Jarrett, Elizabeth F. Lee, Cory Duprey, Russel J. Reiter, Andrzej T. Slominski

**Affiliations:** 1 Department of Dermatology, University of Alabama at Birmingham, Birmingham, AL USA; 2 Department of Pathology and Laboratory Medicine, University of Tennessee HSC, Memphis, TN USA; 3 Department of Toxicology and Cancer Biology, The Markey Cancer Center, College of Medicine, University of Kentucky, Lexington, KY USA; 4 Department of Cellular & Structural Biology, UT Health, San Antonio, TX USA; 5 Comprehensive Cancer Center, Cancer Chemoprevention Program, and Nutrition Obesity Research Center, University of Alabama at Birmingham, Birmingham, AL USA; 6 VA Medical Center, Birmingham, AL USA

## Abstract

Ultraviolet light (UV) is an inducer of reactive oxygen species (ROS) as well as 6-4-photoproducts and cyclobutane pyrimidine dimers (CPD) in the skin, which further cause damage to the skin cells. Irradiation of cultured human melanocytes with UVB stimulated ROS production, which was reduced in cells treated with melatonin or its metabolites: 6-hydroxymelatonin (6-OHM), N1-acetyl-N2-formyl-5-methoxykynuramine (AFMK), *N*-acetylserotonin (NAS), and 5-methoxytryptamine (5-MT). Melatonin and its derivatives also stimulated the expression of NRF2 (nuclear factor erythroid 2 [NF-E2]-related factor 2) and its target enzymes and proteins that play an important role in cell protection from different damaging factors including UVB. Silencing of NRF2 using siRNA diminished the protective effects of melatonin, while the membrane melatonin receptors (MT1 or MT2) did not change the activities of either melatonin or its derivatives. Melatonin and its metabolites enhanced the DNA repair in melanocytes exposed to UVB and stimulated expression of p53 phosphorylated at Ser-15. In conclusion, melatonin and its metabolites protect melanocytes from UVB-induced DNA damage and oxidative stress through activation of NRF2-dependent pathways; these actions are independent of an effect on the classic membrane melatonin receptors. Thus, melatonin and its derivatives can serve as excellent protectors of melanocytes against UVB-induced pathology.

## Introduction

Exposure of the skin to ultraviolet radiation (UVR), especially UVB (290–320 nm), absorbed mainly in the epidermis, induces direct DNA damage^1^ as cyclobutane pyrimidine dimers (CPD) and pyrimidine photoproducts (6–4)PPs^2, 3^ with additional production of reactive oxygen species (ROS)^4^. Collectively, these changes have detrimental effects that include carcinogenesis, cell senescence and other skin pathologies. While nucleotide excision repair (NER) mechanism is responsible for a repair of photoproducts, removal of ROS or converting them to a less toxic product depends on enzymes and proteins including: heme-oxygenase, glutathione peroxidase, catalase, gluthatione, etc.^5^. The enzymes and proteins responsible for antioxidative response are under the control of nuclear factor erythroid 2 –like 2 (NRF2). In response to oxidative stress and ROS, NRF2 is released from Keap (Kelch- like ECH-associated protein), translocates to the nucleus, binds to ARE (anti-oxidant response element) and further activates detoxifying enzymes^6^.

Due to their constant exposure to oxidative stress caused by different environmental factors, skin cells express high levels of NRF2 that protects the skin^7^. NRF2 activation has been a target in treatment of many chronic diseases^8^. Melanocytes produce melanin that protects the skin from deleterious effects of UV. Studies suggest that the susceptibility of melanocytes to oxidative damage is increased due to active melanogenesis^9^ and increased levels of ROS^10^.

Melatonin (N-acetyl-5-methoxytryptamine) protects keratinocytes from detrimental UV radiation^11^ and a plethora of other mammalian cells against oxidative stress^12–14^. Melatonin a is produced primarily in pineal gland and retina^15^, but also at extrapineal sites^16^, including human skin^17, 18^ and other peripheral organs^19^. Melatonin, in addition of modulating circadian rhythms^20^, also exhibits antioxidant, anti-inflammatory, antiproliferative and prodifferentiation activities^21, 22^. Melatonin actions are mediated through its cognate membrane bound type 1 and 2 (MT1 and MT2) receptors or through receptor-independent mechanisms^23^. Metabolites of melatonin can also function as potent antioxidants^24^ or pro-oxidants^25^. They also act as free radical scavengers or inducers of anti-oxidative enzymes^12, 13, 26^. Indolic, kynurenic, and classical pathways are described as the main pathways of melatonin metabolism^12, 17, 27^ and can also be produced in skin under UVB exposure^28^. N-acetylserotonin (NAS) is produced from serotonin and serves as a melatonin precursor^29, 30^, but it is also a melatonin metabolite^17^. N1-acetyl-N2-formyl-5-methoxykynuramine (AFMK) is produced from melatonin via kynuric pathway^12, 27^, through interaction with H_2_O_2_^26, 31^, or due to UVB exposure^26^. Melatonin metabolites also include 6-Hydroxymelatonin (6-OHM) and 5-methoxytryptamine (5-MT) produced via classical or indolic pathway, respectively^11^.

Since melatonin can affect the phenotype of normal human melanocytes^32^, herein we tested whether its direct or indirect activities include protection of epidermal melanocytes against UVB-induced damage. Potential receptor-mediated and independent mechanism of action were also evaluated. Since it was proposed that NRF2 acts as a master regulator of antioxidative responses and protection against UV^6^, we investigated whether protective effects of melatonin and its metabolites in human melanocytes are mediated through activation of NRF2.

## Results

### Melatonin and its metabolites protect melanocytes from UVB- induces oxidative stress

**Figure 1:**
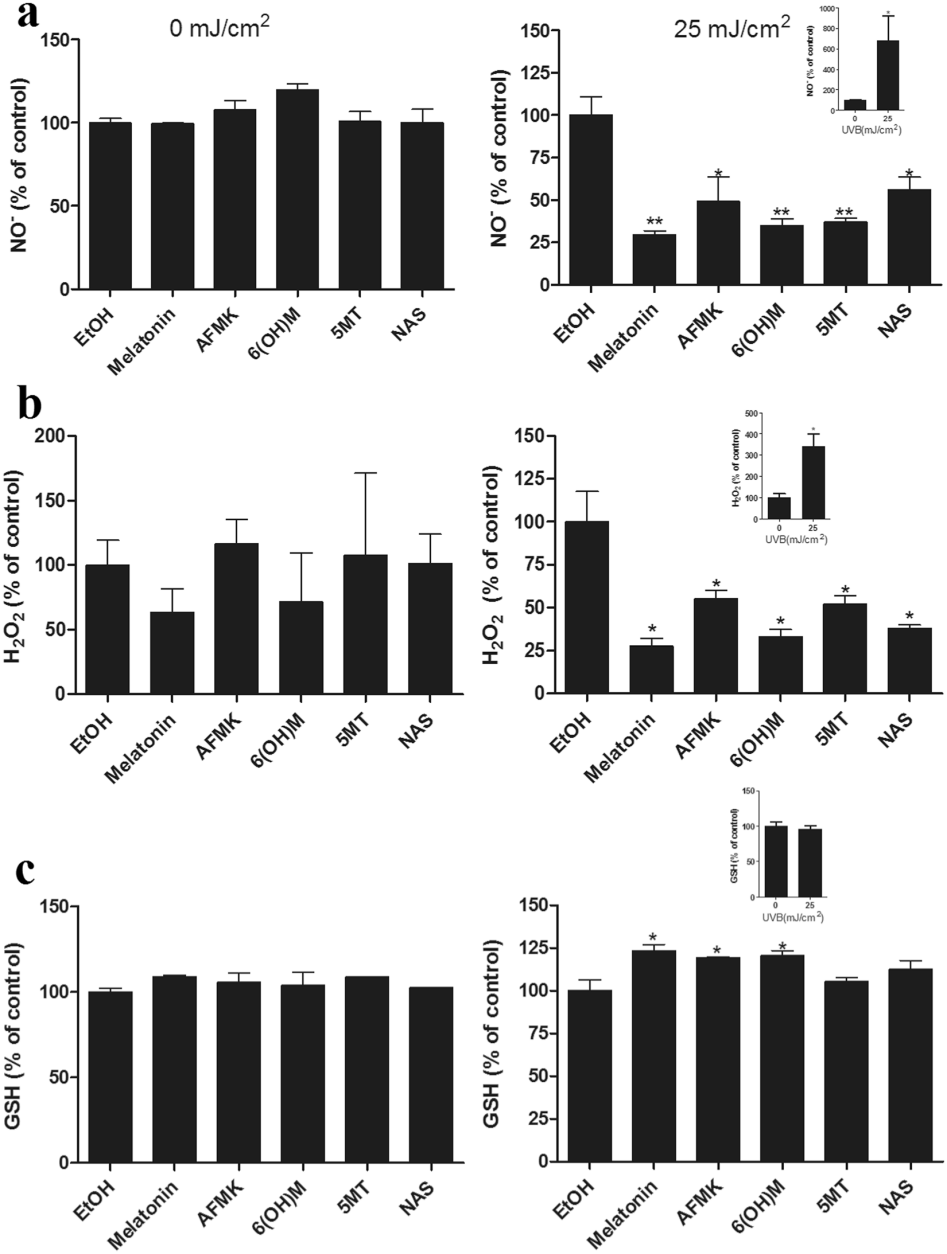
Melatonin, NAS, 6-OHM, AFMK, and 5-MT reduce UVB-induced oxidative stress. UVB-irradiated (25 mJ/cm^2^) and non-irradiated melanocytes (comparison presented as inserts) were treated with melatonin or its metabolites for 24 h prior UV irradiation at the concentration 5 × 10^−5^ M. NO-(**a**) or H_2_O_2_ (**b**) produced in melanocytes were determined 30 min after UVB irradiation, while GSH levels (**c**) were determined 1 h post UVB. Data were analyzed using student t-test, *p < 0.05, **p < 0.01.

Skin cells exposed to UVB produce ROS and suffer DNA damage^4^. Melatonin can prevent the harmful effects of UVB on keratinocytes^33^, as do melatonin metabolites: AFMK, 5-MT, 6-OHM and NAS^24^. We assessed the protection from oxidative stress and antioxidative defence mechanisms in cells treated with melatonin or its metabolites that were irradiated with UVB intensities of 25 or 50 mJ/cm^2^; this dose does not significantly affect the survival of melanocytes^34^. Melatonin protects cells from toxic effects of NO^35^ or H_2_O_2_^31^. To evaluate the protective effects against oxidative damage, normal human epidermal melanocytes (HEMn) were treated with melatonin or its metabolites for 24 h. After incubation, cells were exposed to UVB intensities of 0 or 25 mJ/cm^2^ and NO or H_2_O_2_ were recorded 30 min later. Figure 1 shows significant reductions in NO (a) and H_2_O_2_ (b) directly produced in UVB-irradiated cells treated with melatonin or its metabolites; melatonin and its derivatives had similar protective actions. Non-irradiated cells showed no changes in NO or H_2_O_2_ when compared to control (Fig. 1a and b), while the levels of both NO or H_2_O_2_, increased in irradiated cells (Fig. 1. inserts a and b). Melatonin, 6-OHM and 5-MT were the strongest NO reducers, followed by AFMK and NAS. All metabolites showed similar effects as inhibitors of H_2_O_2_. GSH levels are lowered following UVB irradiation^36^, which was thwarted by melatonin, as described in human vascular endothelial cells^37^ and keratinocytes^24^. Melatonin or its metabolites were added to the melanocytes before the irradiation. The levels of GSH were recorded 1 h after the cells were exposed to UVB. As shown in Fig. 1c (insert), a UVB intensity of 25 mJ/cm^2^ did not significantly reduce the level of GSH as anticipated, because of the melanogenic activity of melanocytes. Melanin synthesis in melanocytes consumes cysteine^38^, which is also an important component of GSH; therefore under the influence of UVB there is an inadequate response in terms of oxidative damage thereby preserving GSH levels in melanocytes^9^. However, relative GSH levels rose after treatment with melatonin, AFMK or 6-OHM, while 5-MT and NAS had no effect on GSH in UVB exposed cells (Fig. 1c).

### Melatonin and its metabolites reduce UVB- induced DNA damage in melanocytes

**Figure 2:**
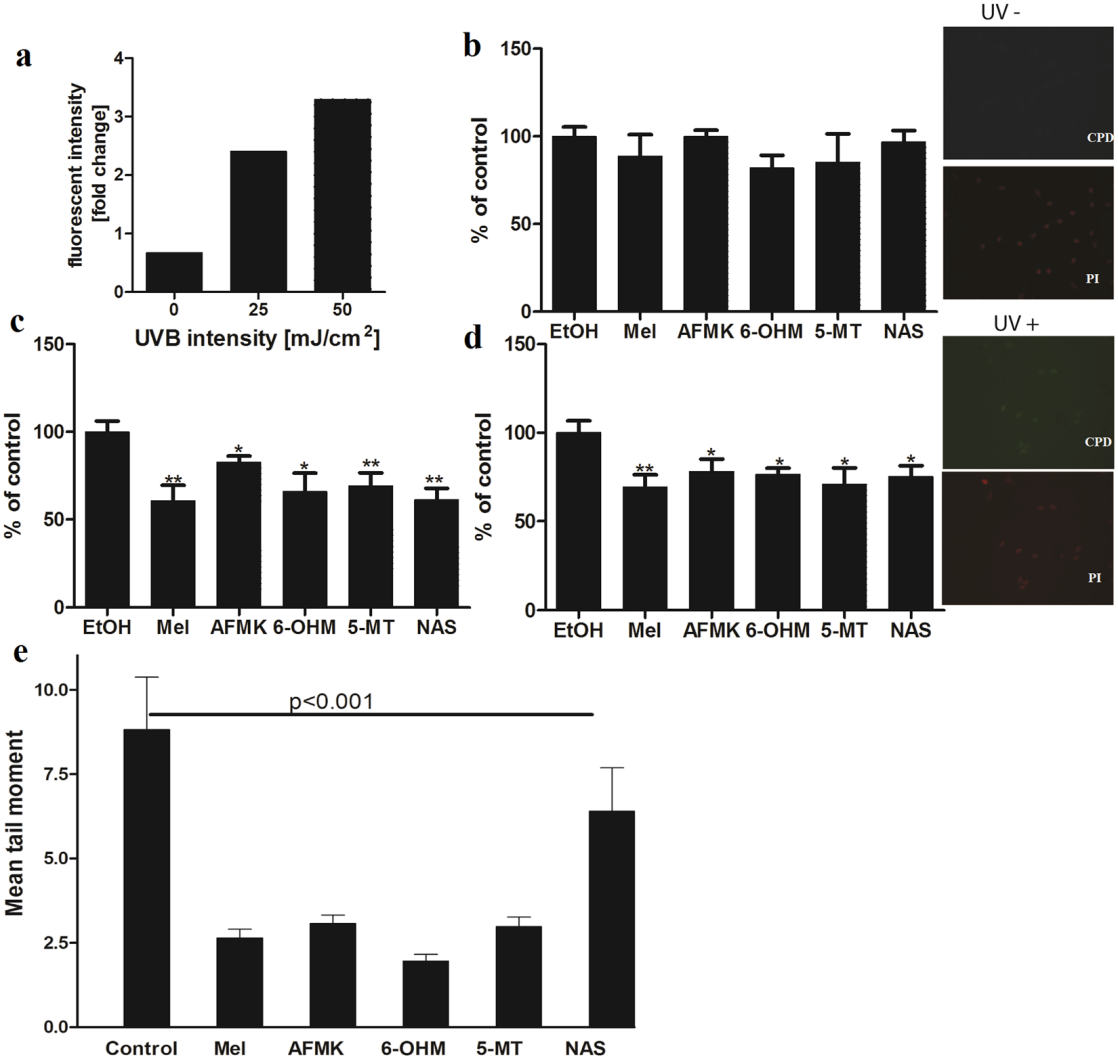
Melatonin, NAS, 6-OHM, AFMK, and 5-MT exhibit repair capacities in UVB-induced DNA damage in treated melanocytes as shown by reduced levels of CPD and comet cell tail moments. Cells were treated with melatonin or its metabolites for 24 h prior UV irradiation at the concentration 5 × 10^−5^ M, further exposed to UVB intensities of 25 or 50 mJ/cm^2^ and immediately treated again with melatonin or its derivatives for 3 h. Cells were fixed and stained with anti-CPD antibody and further imaged with a fluorescence microscope. Fluorescence intensity was analysed using ImageJ software and data were analysed using Graph Pad Prizm. CPD formation under different UVB intensity levels are shown in (**a**). Figure presents CPD levels after 0 mJ/cm^2^ (**b**), 25 mJ/cm^2^ (**c**) and 50 mJ/cm^2^ (**d**). Data are presented as percentile of control and were analyzed using t- test, *p < 0.05, **p < 0.01. As an example, images of the melanocytes stained with CPD antibody (green) and nuclear staining with propidium iodine (red) show no CPD signal in non-irradiated melanocytes (UV−) and positive CPD signal in UVB (25 mJ/cm^2^) irradiated cells (UV+) (magnification 20x). Also, melanocytes were treated with melatonin or its metabolites before and after UV irradiation (200 mJ/cm^3^). Tail moment is an index of DNA damage. Data are analysed using student t-test and p < 0.001 for all conditions (**e**).

UVB induces DNA damage in cells. The DNA repair capacity of melatonin and its metabolites was measured as a reduction in CPDs in HEMn exposed to UVB. Immediately after irradiation, cells were treated with melatonin or its derivatives. CPD-positive cells were labelled with CPD-specific antibody and scored under the microscope. As shown in Fig. 2a, UVB irradiation increased production of CPD (Fig. 2, inserted pictures). Treatment with melatonin or its metabolites caused obvious reduction of CPD levels in cells exposed to UVB 25 mJ/cm^2^ (Fig. 2c) or 50 mJ/cm^2^ (Fig. 2d); this contrasted with the lack of such an effect in control cells (Fig. 2b). Melatonin reduced CPD levels by approximately 40%. While 5-MT and NAS showed a potency similar to melatonin in cells exposed to 25 mJ/cm^2^, a slightly weaker effect was noted in cells exposed to 50 mJ/cm2. AFMK and 6-OHM were somewhat less potent than melatonin, but still effective. To confirm the above findings we used comet assay as previously described^39^. Thus, we exposed cells to 200 mJ/cm^2^ UVB; to measure the protective effects of melatonin and its metabolites we treated cells with the mentioned compounds 24 h before the exposure to UVB and for an additional 3 h after. Melatonin, AFMK, 6-OHM, 5-MT and NAS all significantly reduced the tail moment of the comet images (p < 0.001) in comparison to the non-treated cells (Fig. 2e).

**Figure 3:**
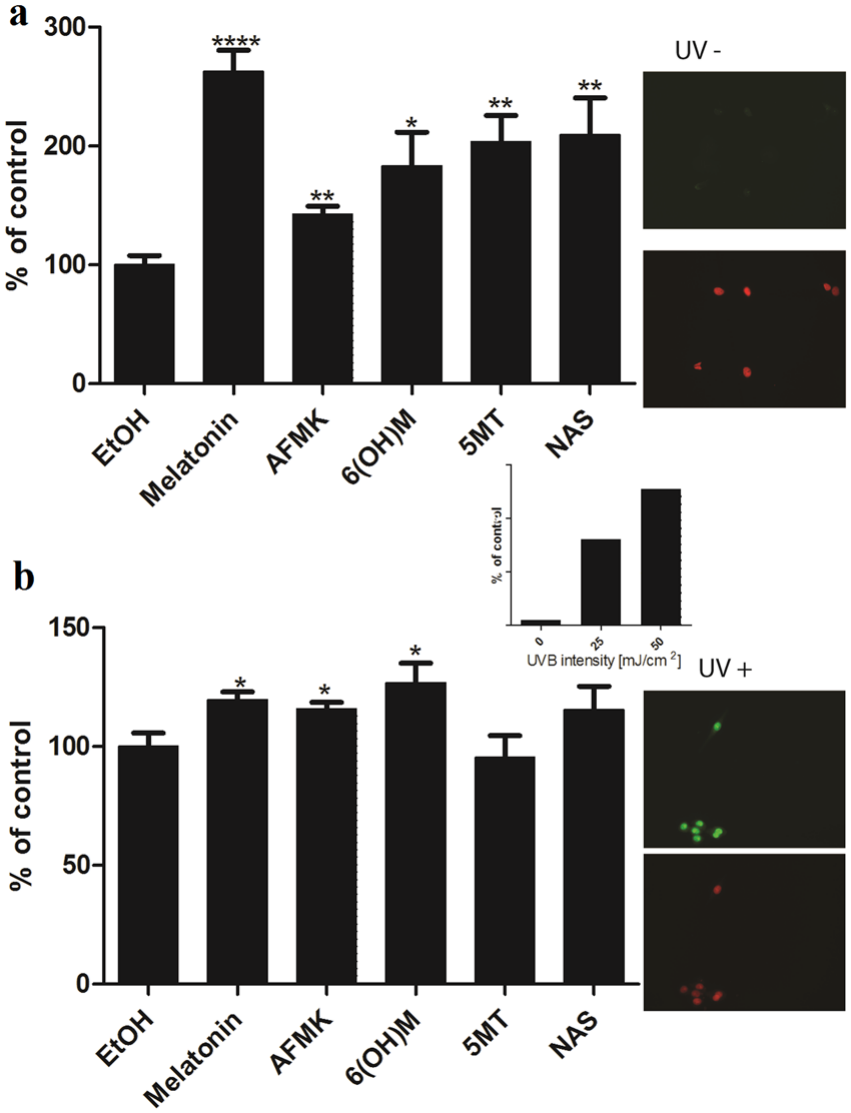
UVB irradiation intensifies p53 levels. Melatonin, NAS, 6-OHM, AFMK, and 5-MT treated HEMn elevated levels of p53 phosphorylated at Serine 15 after UVB exposure. Melanocytes were treated with melatonin or its metabolites for 24 h before UVB exposure. Cells were exposed to UVB intensities of 25 (**a**), or 50 mJ/cm^2^ (**b**) and immediately treated again with melatonin or its derivatives for 12 h. Cells were further fixed and stained with anti-phosphorylated p53S15 antibody. Stained cells were imaged with a fluorescence microscope; fluorescence intensity was analysed using ImageJ software. P53 levels increased proportional to UVB intensity (insert). Data are analysed using Graph Pad Prizm. Data are presented as percentile of control and were analyzed using t- test, *p < 0.05, **p < 0.01, ***p < 0.001.

UVB induces tumor suppressor factor p53, as a response to DNA damage^40^. P53 further accumulates in the nucleus and activates the DNA repair process^41^. Melatonin is known to induce phosphorylation of p53 at Ser-15^42, 43^, thus activating p53. We tested the ability of melatonin and its metabolites to induce p53 phosphorylation at Ser-15 as a response to UVB damage in comparison to melatonin. Melanocytes were treated for 3 h with melatonin or its metabolites after UVB exposure. All molecules tested significantly enhanced the expression of Ser-15 phosphorylated p53 (Fig. 3). The intensity of UVB was directly proportional to p53 accumulation (Fig. 3 inserts). Melatonin enhanced the p53 rise in cells exposed to 25 mJ/cm^2^ of UVB; p53 more than doubled with melatonin having the most robust effect of all metabolites, although metabolites were also potent (Fig. 3a). The induction of p53 under the influence of melatonin, AFMK and 6-OHM was also detected at higher UVB intensity, but to a lesser extent (Fig. 3b). In contrast, 5-MT and NAS failed to augment the expression of Ser-15 p53 under the described conditions (Fig. 3b).

**Figure 4:**
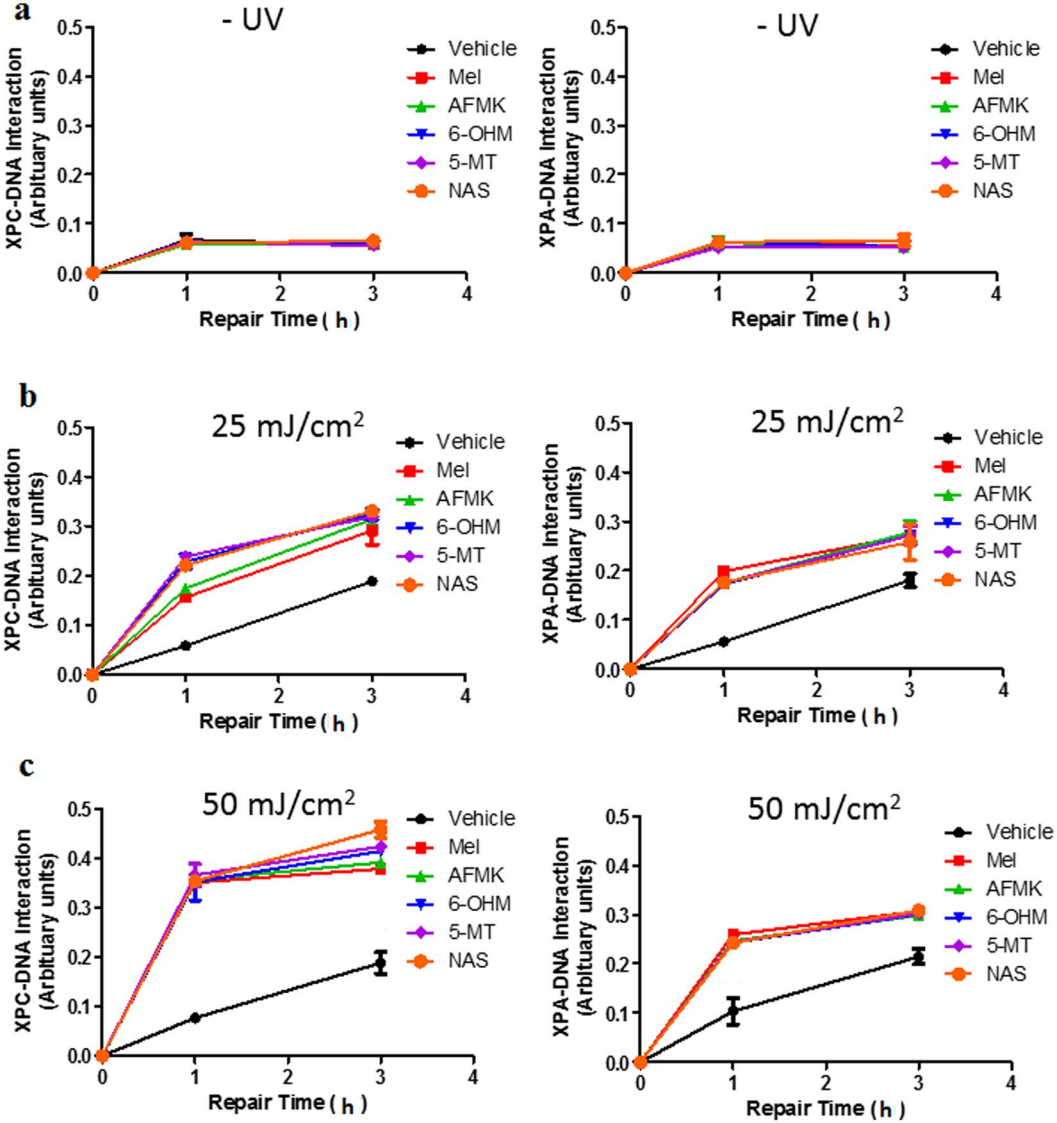
Melatonin and its metabolites enhance the XPC- and XPA-DNA-interaction. Melanocytes were treated with melatonin or its metabolites for 24 h in complete melanocyte medium. Cells were further exposed to either (**a**) non UVB-treated (0 mJ/cm^2^) or (**b**) 25 mJ/cm^2^ or (**c**) 50 mJ/cm^2^. Synthetic oligonucleotides (Molecular Beacons) were assembled to form 5′-biotinylated duplex DNA fragment. The substrate provides a substrate for NER proteins and DNA-protein interactions were determined using anti-XPC and anti-XPA antibodies as described in the Methods. Treatments significantly different from vehicle were determined by one-way ANOVA; *p ≤ 0.05. All treatments in B or C were statistically significant when compared to vehicle.

To further interrogate the role of melatonin and its metabolites to directly impact nucleotide excision repair (NER), we utilized a technique termed Oligonucleotide retrieval immunoprecipitation (ORiP)^44^. The ORiP assay relies on UV exposure or mock treatment of cells, isolation of nuclear fractions, incubation with a biotinylated oligonucleotide construct containing a UV-damaged fragment, retrieval of the oligonucleotide by streptavidin and identification of bound proteins. Nuclear fractions isolated from UV-damaged cells demonstrated binding of NER complex containing two core factors of this repair pathway: Xeroderma pigmentosum, complementation group C (XPC) and Xeroderma pigmentosum, complementation group A (XPA) proteins to photoproduct containing-substrate (Fig. 4). Further, pre-treatment of either melatonin or one of its metabolites significantly enhanced the XPC and XPA interactions with the DNA substrate (compared to UV treatment alone; P ≤ 0.05) with both treatments of 25 mJ/cm^2^ or 50 mJ/cm^2^ of UVB (Fig. 4). These results indicate that melatonin and its metabolites can induce repair of DNA damaged by UVB in HEMn.

### Melatonin and melatonin metabolites antioxidative properties are MT receptor independent

**Figure 5:**
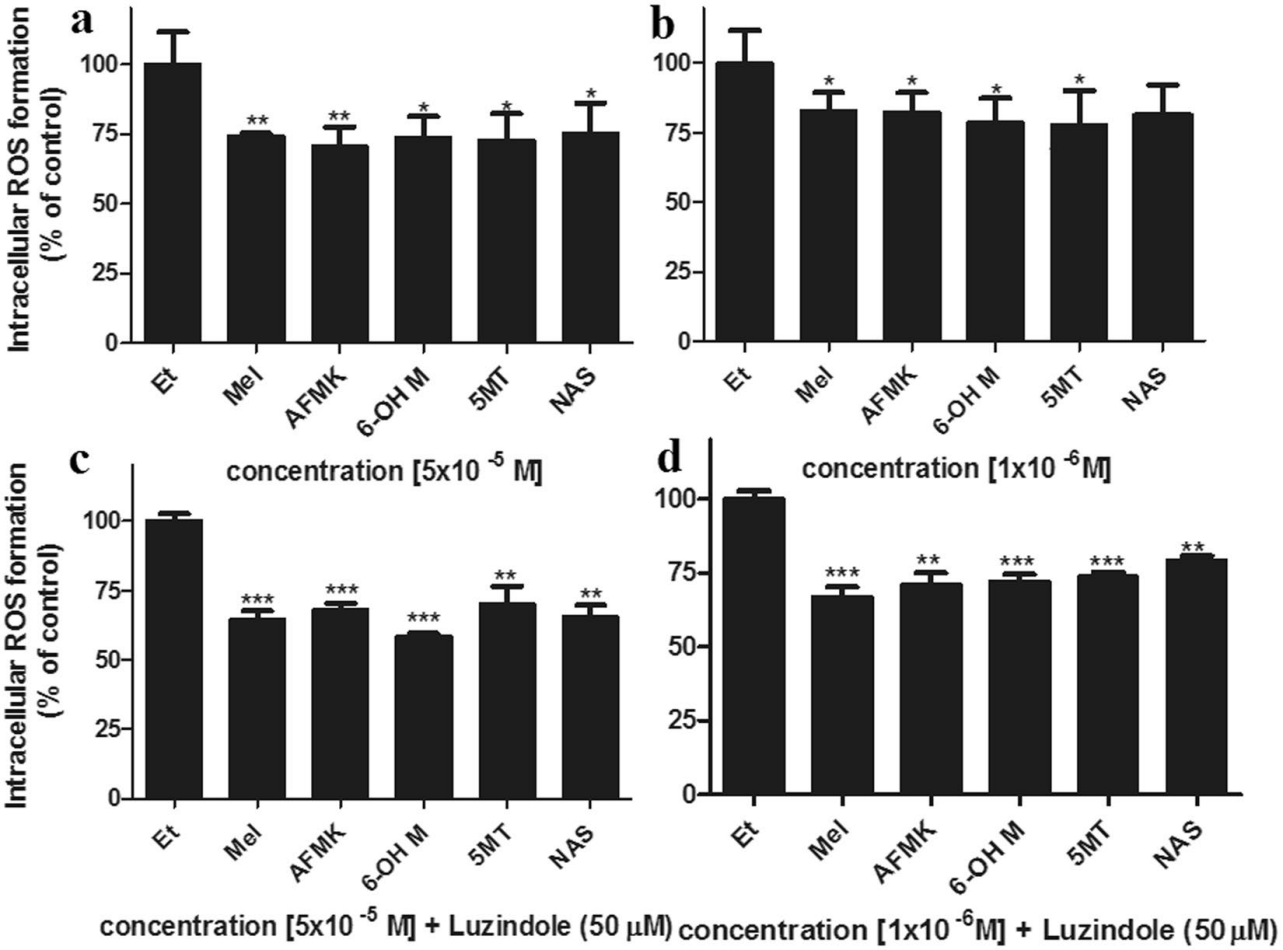
Melatonin, NAS, 6-OHM, AFMK, and 5-MT reduce levels of ROS produced in UVB-irradiated melanocytes. Cells were treated without (**a**,**b**) or with (**c**,**d**) luzindole 1 h prior treatment with melatonin, AFMK, 6-OHT, 5-MT, NAS, or EtOH for 1 h at the concentrations of 5 × 10^−5^ M (**a**,**c**) or 1 × 10^−6^ M (**b**,**d**). CM-H2DCFDA dye was added to the cells for 30 min and cells are further irradiated with 50 mJ/cm^2^ of UVB. ROS levels in melanocytes were determined 30 min after UVB irradiation. The differences in H_2_O_2_ production under different UVB intensities are shown as insert. Data are presented as percent of control and were analyzed using t-test, *p < 0.05, **p < 0.01, and ***p < 0.001.

Melatonin and its metabolites serve as local antioxidants under oxidative stress conditions caused by various environmental factors, including UVR^11^. The exposure to UV promotes the expression of MT1 and MT2 melatonin receptors in skin^11, 45, 46^. Hence, we evaluated whether melatonin’s protective effects are mediated by membrane bound melatonin receptors. HEMn were treated with melatonin or its metabolites at different concentrations in the absence or presence of luzindole, a melatonin receptor antagonist^47^. Melatonin and its metabolites exerted protective effects against UVB (50 mJ/cm^2^) induced ROS formation even in the presence of luzindole (Fig. 5). The effects of melatonin, AFMK and NAS were dose depended, being more pronounced at the higher concentration (Fig. 5a and b). Melatonin and AFMK significantly decreased ROS levels at different concentrations, 5 × 10^−5^ M (p < 0.01) (a) and 1 × 10^−6^ M (p < 0.05) (b). Other metabolites, 6-OHM and 5-MT showed no concentration dependency (p < 0.01), while NAS had an effect on ROS only at 5 × 10^−5^ M. The protective effects of melatonin and its metabolites at concentrations (5 × 10^−5^ M or 1 × 10^−6^ M) were not affected by luzindole (Fig. 5c and d). To corroborate whether or not the antioxidative effects of melatonin and its metabolites are membrane bound receptor independent, we tested the levels of both melatonin receptors (MT1 and MT2). The results showed very low levels of MT receptors in melanocytes. Their detection required immunoprecipitation of the antigen prior Western blot analysis, which otherwise was negative when whole extracts were probed with the antibodies (Supplementary Fig. S1).

### Melatonin and its metabolites induce the expression of antioxidative enzymes in melanocytes

To further evaluate the status of oxidative stress in UVB irradiated melanocytes we examined the expression of major antioxidant enzymes, including catalase (CAT), glutathione peroxidase (GPx), glutathione reductase (GR), glutathione-S-transferase (GSTP1), superoxide dismutase (Cu/Zn-Sod and Mn-SOD), glutamylcysteine synthetase (GCS), and NAD(P)H dehydrogenase, quinine 2 (NQO2) in melanocytes. As shown in Supplementary Fig. S2, at the RNA level, melatonin and its metabolites induced antioxidant enzymes after cells were exposed to UVB. The stimulation was dependent on the intensity of UVB; the stronger the intensity of UVB the greater were the effects of melatonin or its metabolites. The NRF2 transcription factor induces antioxidant genes; therefore, we have tested the expression of NRF2 and other related genes (HO-1, GCS and GSTP1) relative to the protective of melatonin or its metabolites in response to UVB induced oxidative stress. Melatonin exhibited a discernible effect on NRF2 expression. NRF2 levels were 4–5 fold higher in cells treated with melatonin regardless of UVB treatment. All other metabolites were also strong NRF2 inducers (4–6 times when compared to control), while 5-MT was somewhat more potent than the others. As anticipated, HO-1, was also strongly induced by melatonin or its metabolites after treatment with UVB. Melatonin induced HO-1 levels remarkably 65 times or 4 times when compared to control after treatment with 75 mJ/cm^2^ or 50 mJ/cm^2^, while no effect was observed after 25 mJ/cm^2^. Surprisingly, the levels of HO-1 dropped after treatment with melatonin in cells not exposed to UVB. GCS levels were strongly induced after treatment with melatonin or its metabolites in UVB-irradiated cells (Supplementary Fig. S2). The levels of GCS ranged from 15–35 times more when compared to control in cells treated with 75 mJ/cm^2^ UVB, with melatonin being the most potent. A 3–5-fold increase in GSC levels were detected after treatment with 50 mJ/cm^2^ of UVB, with NAS being more potent then melatonin or other metabolites. Similarly, GSTP1 levels were also strongly induced by melatonin and metabolites (Supplementary Fig. S2).

### Melatonin protective action from oxidative is through NRF2 pathway

**Figure 6:**
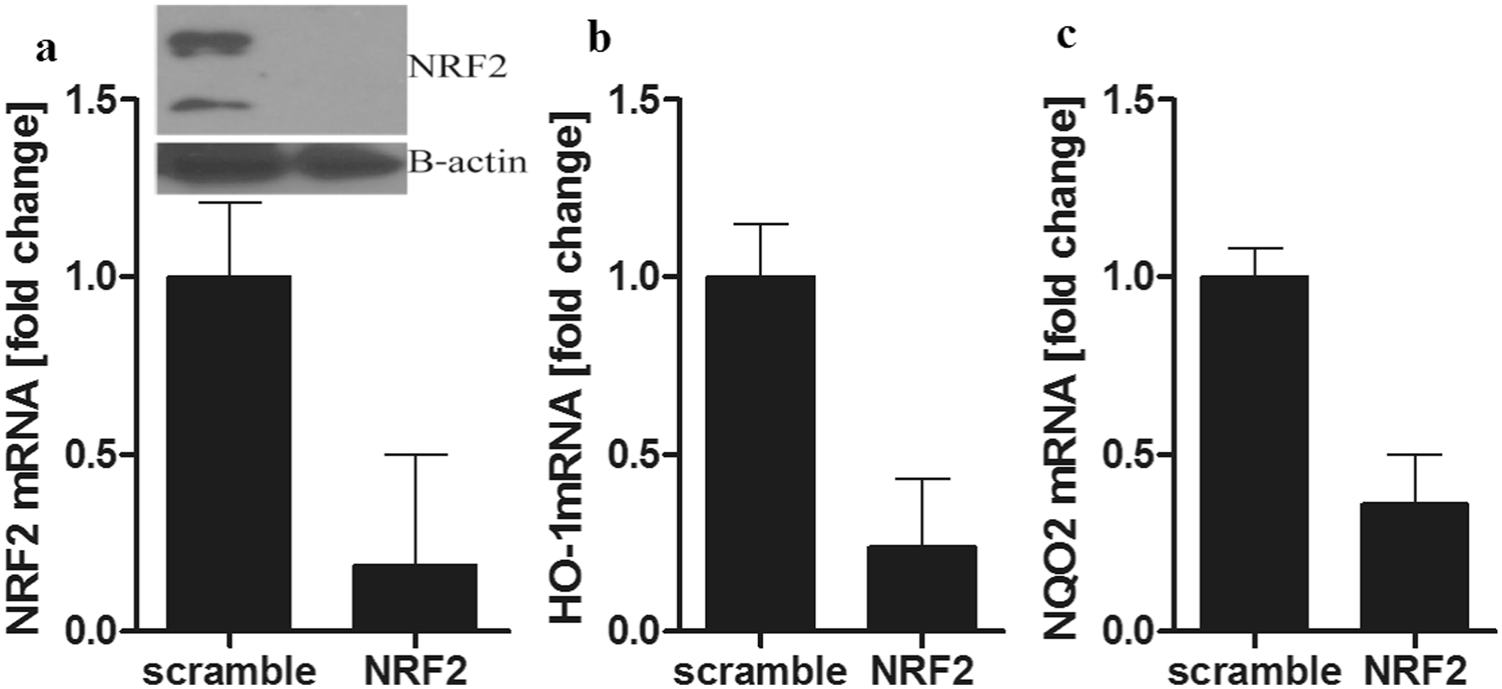
Silencing of the NRF2 in human melanocytes reduced the effect of melatonin on the expression of NRF2 and NRF2-related genes: HO-1 and NQO2. Melanocytes were transfected with scrambled or NRF2 siRNA and treated with 5 × 10^−5^ M melatonin. Cells were lysed after treatment and total RNA extracted. NRF2 mRNA (**a**), HO-1 mRNA (**b**) and NQO2 (**c**) levels were measured using reagents for RTPCR and normalized relative to B-actin RNA. Levels of NRF2 and B-actin were assessed 72 h after transfection with NRF2 or scrambled siRNA, by western blotting of whole-cell extracts (insert).

**Figure 7:**
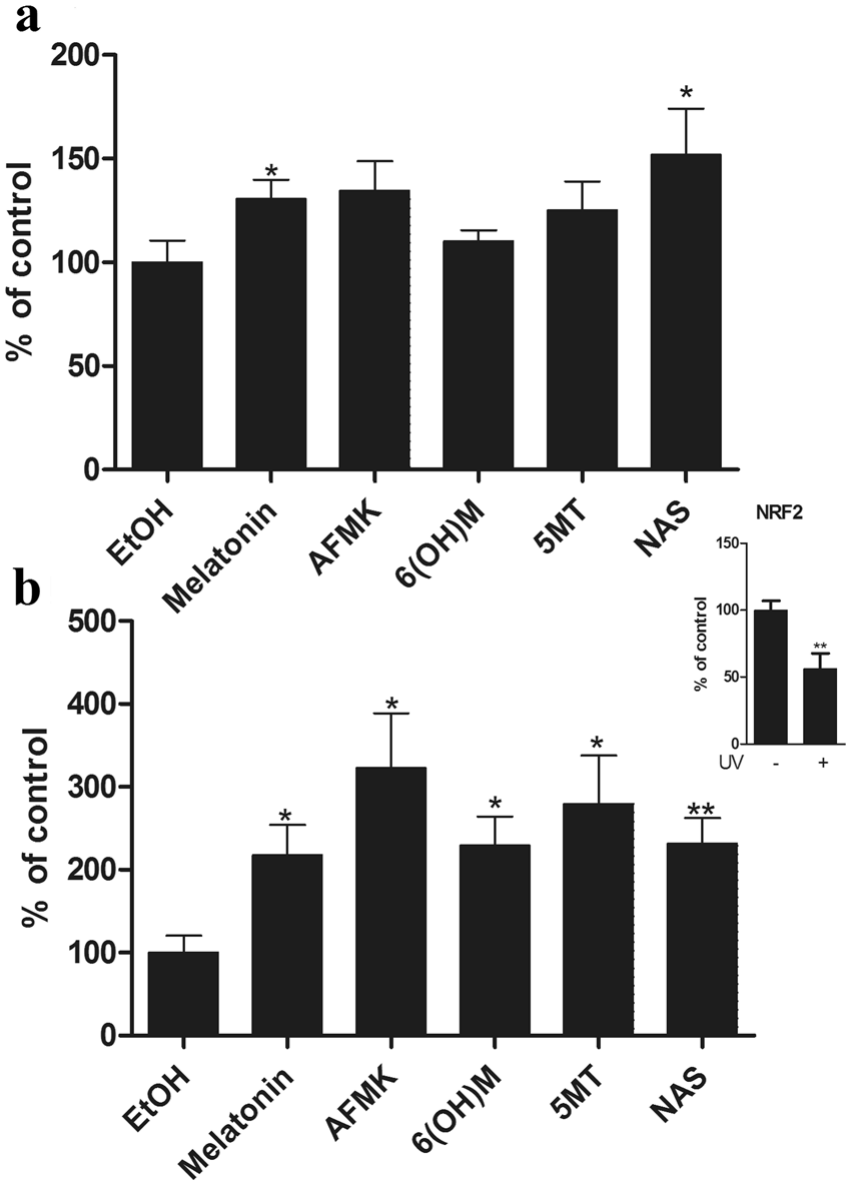
Melatonin, NAS, 6-OHM, AFMK, and 5-MT treated melanocytes increased NRF2 expression after UVB exposure. Melanocytes were exposed to UVB intensities of 0 or 50 mJ/cm^2^ and immediately treated with melatonin or its derivative for 24 h. Cells were further fixed and stained with anti-NRF2 antibody. Stained cells were imaged with a fluorescence microscope, fluorescence intensity was analysed using ImageJ software, and data are analysed using Graph Pad Prizm after 0 mJ/cm^2^ (**a**) and 50 mJ/cm^2^ (**b**). In addition, UVB diminishes NRF2 expression (insert). Data are presented as percentile of control and were analyzed using t- test, *p < 0.05, **p < 0.01.

To investigate the potential mechanism of melatonin in protection from oxidative stress caused by UVB in connection to NRF2, we used NRF2 interfering RNA (NRF2 siRNA) to downregulate NRF2 expression. Figure 6a shows the RT-PCR results indicating that mRNA level of NRF2 in cells was reduced to more than half in comparison to control. Cells were also treated with melatonin. Although we have shown that melatonin induces NRF2 (Supplementary Fig. 2), the silencing of NRF2 abrogated the effects of melatonin, while cells treated with scrambled siRNA showed no change in NRF2 mRNA (Fig. 6a) or protein levels (Fig. 6 insert). To determine whether antioxidative genes downstream of NRF2-ARE pathway are affected by silencing of NRF2, we examined the HO-1 and NQO2 on mRNA. Figure 6 shows the reduction of the main phase II detoxifying enzymes: HO-1 (b) and NQO2 (c) in the cells after silencing NFR2 and treatment with melatonin when compared to scrambled siRNA. Immunofluorescent analysis on melanocytes treated with melatonin or its metabolites after exposure to UVB revealed greater intensity of NRF2 in comparison to non-irradiated cells (Fig. 7). Melatonin and NAS slightly enhanced NRF2 expression in non-irradiated cells (Fig. 7a), but all metabolites induced NRF2 after UVB exposure more than double when compared to control (Fig. 7b), with NAS having the strongest influence. Cells exposed to UVB showed reduction in NRF2 levels (Fig. 7 insert), which is in accordance with previous reports^48^.

## Discussion

Skin and skin cells are under the constant influence of different environmental stressors, including UVB, which can induce formation of reactive oxygen species (ROS) and cause oxidative damage^4, 49^. The damage to the skin results in cell senescence, photoaging, carcinogenesis, inflammation, etc. Increased levels of ROS after exposure to UVB has toxic effects on melanocytes, as described in vitiligo^50^. Natural compounds, such as melatonin, have the potential of activating endogenous antioxidants defence mechanisms and enhancing protection of skin from UV^12^. Melatonin is antioxidant and a free radicals scavenger^14^; it detoxifies NO^35^, H_2_O_2_ and O_2_•−^31^. Studies have shown the potential beneficial effects of melatonin in treatment of many chronic diseases while it exhibits low or absent toxicity^51^. In addition, in animal models, melatonin displays protective effects that are NRF2-mediated^52^.

Herein, primary human melanocytes were treated with pharmacological doses of melatonin or its derivatives. We have demonstrated that melatonin, as well as its metabolites (AFMK, 6-OHM, 5-MT, and NAS) protect cells from UVB-induced oxidative stress, in accordance with previous studies^24, 33^. The exposure of melanocytes to UVB resulted in formation of intracellular ROS, including NO and H_2_O_2_, as well as the reduction of intracellular glutathione. Pretreatment of melanocytes with melatonin or its metabolites protect against cellular damage caused by ROS. They all reduced the levels of ROS (Fig. 5), especially NO and H_2_O_2_ (Fig. 1). This process does not involve membrane bound melatonin receptors, since melatonin and its metabolites exhibit their antioxidative effects even after membrane melatonin receptors were blocked by the receptor antagonist, luzindole (Fig. 5). Melatonin receptors (MT1 and MT2) are known to be expressed in human epidermal melanocytes at very low levels (Supplementary Fig. S1). Melatonin also promoted glutathione production, which serves as a first line of cellular defence against oxidative damage. We have shown that melatonin, AFMK and 6-OH, excluding 5-MT and NAS, exhibit slight GSH activation (Fig. 1), while stimulating genes encoding GSH production (Supplementary Fig. S2). Despite the correlation between melanin and ROS levels, the latter accumulate and subsequently reduce cellular glutathione concentrations in human melanocytes as previously reported^9^; clearly melatonin plays an important role in glutathione regulation^37, 53^.

Oxidative stress can impair the main repair pathway for UVB-induced DNA damage^2, 3^. Direct UVB-induced DNA lesions (CPD) were present in melanocytes exposed to UVB (Fig. 2a). CPD formation noticeably decreased in UVB-irradiated melanocytes treated with melatonin or its metabolites (Fig. 2c,d). Also, melatonin and its metabolites enhanced UVB-induced DNA repair (Fig. 2e). Tumor suppressor protein p53 is another important regulator of oxidative stress in skin cells. Activation of p53 is one means of reducing oxidative stress in melanocytes^10^ resulting from different stressors, including UVB^40^. Phosphorylation of p53 is important for activation of DNA repair in cells^54^, especially at the Ser-15 site responsible for DNA damage prevention^41^, which is stimulated by melatonin^23, 55^. Melatonin and its metabolites augmented the accumulation of UVB-induced p53 in the nucleus by increasing phosphorylation of p53 on Ser-15 in melanocytes (Fig. 3), as well as enhanced nucleotide excision repair via enhanced interactions between damaged DNA and the NER core factors XPC and XPA (Fig. 4), indicating the protective actions of these compounds against UVB induced cell damage, which is in accordance with previous results^24^.

We have confirmed (Fig. 7, insert) what has been previously described, i.e., UVB reduced the expression of NRF2 in melanocytes^7, 48^. This downregulation was counteracted by treatment with melatonin and its metabolites (Fig. 7a,b). Melatonin has often been described as a regulator of NRF2^56^. Melatonin and its metabolites also stimulate mRNA expression of antioxidant enzymes and proteins, including catalase, superoxide dismutase, glutathione, NAD(P)H dehydrogenase, glutamylcysteine synthetase and heme oxygenase (Supplementary Fig. S2). In this study we have demonstrated that melatonin, as well as its metabolites (AFMK, 6-OHM, 5-MT, and NAS) induce NRF2 downstream genes and proteins which further protect primary human melanocytes from UVB-induced oxidative stress, similar to their protection in other cells^14, 31^. The mechanism of this activation is mediated by upregulation of NRF2 expression and NRF2- dependant pathway (Fig. 7, and Supplementary Figs S2 and S3). When siRNA against NRF2 was used, a downregulation of HO-1 and NQO2 was observed despite the treatment with the melatonin (Fig. 6). We found that NRF2-activation is important in reduction of UVB-induced damage to the skin cells, as shown by others^57, 58^.

Pre-treatment of melanocytes with melatonin or its metabolites conferred protection against UVB mediated ROS damage. Melatonin and its products stimulated NRF2 activation and expression of NRF2 downstream genes and proteins that counteract oxidative stress. Knocking NRF2 gene confirm the mechanism of melatonin in NRF2-mediated antioxidative effects on melanocytes. This study demonstrates the benefits of natural products, i.e., melatonin and its metabolites, in stimulation of NRF2, an endogenous antioxidative pathway, to prevent deleterious effect UVB and UVB-caused oxidative damage. Our data support the protective role of melatonin and it metabolites in antioxidant skin defence mechanism. The current results demonstrate the high protection that melatonin and its metabolites, as natural antioxidants, can provide to skin cells against UVB-induced damage. While there are differences in the potencies of the protection provided by melatonin and its metabolites, these differences are not great given that all melatonin metabolites exhibited strong protective effects against UVB-induced oxidative damage to melanocytes. The present study identifies local melatoninergic system as the protector of melanocytes against UVB-induced pathology, effects that can be amplified by topical application of melatonin or its derivatives.

## Material and Methods

### Chemicals

The source of chemicals is described previously^24^. All chemicals were diluted in pure ethanol. AFMK (*N* ^*1*^-acetyl-*N* ^*2*^-formyl-5-methoxykynuramine) (10005254) was purchased from Cayman Chemical Company (Ann Arbor, MI, USA) (catalog number 10005254), while others were purchased from Sigma-Aldrich (St. Louis, MO): Melatonin (*N*-acetyl-5-methoxytryptamine) (M5250), 6-hydroxymelatonin **(**3-(N-acetylaminoethyl)-6-hydroxy-5-methoxyindole) (H0627), N-acetylserotonin (*N*-acetyl-5-hydroxytryptamine) (A1824), 5-methoxytryptamine **(**2-(5-methoxy-1H-indol-3-yl)ethanamine) (286583).

### Human tissue samples

The experiments were performed in accordance with relevant guidelines (see below) and the experiments were approved by the Institutional Review Board (IRB) at the University of Alabama Birmingham. This protocol was identified as not subject to FDA regulation and not Human Subject Research (IRB protocol N150915001 – Endocrine Functions of the Skin – revised version). Where necessary it is also covered by an exempt protocol category #4 for use of the archival or stored material approved on 7/2/2015 under IRB registration # IRB00000726 under the title “Neuroendocrinology of the skin and other peripheral organs” E150427002. No human subjects were involved in this protocol. However, primary melanocytes were cultured from foreskin that would normally be discarded during surgery. Animal subjects were not included in experimental design.

### Cell culture

Human epidermal melanocytes (HEMn) are isolated from neonatal foreskin of African-American donors and cultured for experiments as previously described^59^. Cells were grown in melanocyte growth media (MGM) supplemented with melanocyte growth factors (MGF) (Lonza Walkersville Inc, Walkersville, MD). For the experiments with UVB the media were replaced with phosphate buffered saline (PBS) prior the exposure. Cells in their third passage were used for the experiments and pretreated with melatonin or its derivatives 24 h before exposure to UVB after UVB exposure for indicated amount of time. It must be noted that melanocytes used for the experiments were darkly pigmented with dendritic morphology that was not affected by UVB doses of 25 and 50 mJ/cm^2^ during relatively short periods of incubation including 3 and 24 h after UVR treatment (Supplemental Fig. S3). UV transilluminator 2000 from Bio-RAD Laboratories was used for UVB irradiation following protocols and UV spectra reported previously^39^.

### Comet assay

DNA damage and repair were assessed using Comet assay, and detecting levels of CPD dimers and p53. The comet assay was performed following the manufacturer’s protocol (Trevigen, Gaithersburg, MD) with experimental design as previously described^39^. Melanocytes were seeded in 12-well plates. Cells were treated with melatonin or its metabolites at the concentration of 5 × 10^−5^ M, or ethanol (vehicle, dilution 5:1,000) for 24 h. Then, the medium was replaced with 1 × PBS and cells were exposed to UVB 200 mJ/cm^2^. PBS was removed and replaced with fresh medium containing compounds and cells were further incubated for 3 h at 37 °C. After detaching, cells were counted and used for the comet assay. Cell suspensions at a density of 1 × 10^5^/ml were combined with molten 1.2% low-melting-point agarose, diluted 1:10, placed onto two frosted slides precoated with 0.6% normal agarose, and incubated at 4 °C for 30 min. Cells were further digested in lysis solution at 4 °C for 60 minutes. DNA strand breaks were separated by electrophoresis in alkaline electrophoreses solution (200 mM NaOH, 1 mM EDTA, pH > 13) in horizontal gel electrophoresis slide tray (Comet-10, Thistle Scientific, UK). DNA breaks were exposed to alkaline unwinding for 40 min at room temperature after which electrophoresis was performed at 25 V for 50 min. Following neutralization in neutralizing buffer (0.4 M Tris, pH7), comets were visualized by propidium iodide (Sigma-Aldrich, St. Louis, MO) staining. The slides were examined and images were captured using Nikon fluorescent microscope and the Leica digital DM 4000b fluorescent microscope equipped with image analysis software. Approximately 60 comet images were taken for each condition. DNA damage was measured by the tail length using Comet Score software available from http://www.scorecomets.com.

### CPD dimers

Following the manufacturer protocol, cells were stained with anti-CPD antibody and nuclei were stained red with propidium iodide as previously described^24^. CPDs in cultured normal melanocytes were visualised using immunofluorescence. Briefly, HEMn were plated onto chamber slides, treated with melatonin or its metabolites at the concentration of 5 × 10^−5^ M, or ethanol (vehicle, dilution 5:1,000) for 24 h prior UVB exposure, and further for 3 h after the exposure. Following the manufacturer protocol, cells were stained with anti-CPD (clone TDM2) (Cosmo Bio Co Ltd., Tokyo, Japan) (dil 1:100) and subsequently with goat anti-mouse IgG-FITC (Santa Cruz Biotechnology, Santa Cruz, CA) (1:100). Nuclei were stained red with Vectashield mounting media with propidium iodide (Vector Laboratories, Burlingame, CA). Stained cells were imaged with a fluorescence microscope. Pictures recorded were analysed using ImageJ software (NIH free download).

NRF2 in cultured normal melanocytes was visualised using immunofluorescence. Briefly, HEMn were plated onto chamber slides, treated with melatonin or its metabolites at the concentration of 5 × 10^−5^ M, or ethanol (vehicle, dilution 5:1,000) for 24 h after UVB exposure. Following the manufacturer protocol, cells were stained with anti-NRF2 (C20) (sc-722, Santa Cruz Biotech., Santa Cruz, CA) (dil 1:50) and subsequently with Alexa-Fluor 488 goat anti rabbit IgG (Invitrogen Molecular Probes, Eugene, Oregon, USA) (1:100). Nuclei were stained red with Vectashield mounting media with propidium iodide (Vector Laboratories, Burlingame, CA). Stained cells were imaged with a fluorescence microscope. Pictures recorded were analysed using ImageJ software (NIH free download).

### Immunofluorescence for p53

Melanocytes were plated onto chamber slides in duplicates. Cells were treated with melatonin, or its metabolites at the concentration of 5 × 10^−5^ M, or ethanol (vehicle, dilution 5:1,000) for 24 h prior UVB exposure and for 3 h for detection of phosphorylated p53 at Ser-15^55^. Cells were further fixed in 4% paraformaldehyde (PFA) for 10 min at room temperature and washed three times with 0.1% Triton X-100 (BioRad, Hercules, CA) in PBS to permebealize membrane. Blocking was performed in 10% FBS in PBS for 1 h at RT after quenching of endogenous peroxidase with 1% H_2_O_2_ (BioRad, Hercules, CA) in PBS. Cells were incubated in phospho p53 Ser-15 (9284 Cell Signaling, Danvers, MA) primary antibodies diluted in blocker 1:100 overnight at 4 °C. The following day cells were washed and incubated with secondary antibody Alexa-Fluor 488 goat anti-rabbit IgG (Invitrogen Molecular Probes, Eugene, Oregon, USA) diluted in blocker 1:100 for 1 h at RT. After washing with PBS, nuclei were stained red with Vectashield mounting media with propidium iodide (Vector Laboratories, Burlingame, CA). Stained cells were imaged with a fluorescence microscope. Pictures recorded were analysed using ImageJ software (NIH free download).

### Reactive oxygen species (ROS) assays

Reactive oxygen species (ROS) assays were used to measure ROS levels in HEMn using a fluorescence method as previously described^24^. Luzindole is used in accordance with the literature^47^. Melanocytes were plated onto 96-well plate. The following day, medium was removed and fresh luzindole (Sigma-Aldrich (St. Louis, MO) was added to the cells at the concentration of 50 µM for 1 h. Cells are further treated with melatonin or its metabolites at the concentrations of 5 × 10^−5^ M, 1 × 10^−6^ M, 1 × 10^−8^ M, 1 × 10^−10^ M, or ethanol (vehicle, dilution 5:1,000) for 1 h. After incubation, cells were washed with cold 1×PBS. CM-H2DCFDA dye (Molecular Probes, Invitrogene, Eugene, Oregon) diluted in HEPES at the 5 µM concentration was added and cells were incubated for additional 30 min. After preincubation with CM-H2DCFDA dye, cells were irradiated with 50 mJ/cm^2^ of UVB and placed in incubator. Thirty minutes post-UVB exposure, cells were washed with PBS. The generation of ROS was determined by measuring the fluorescence at 480 nm excitation and 528 nm emission, on SpectraMax M2e instrument (Molecular Devices, Sunnyvale, CA). Data were presented as percentile of control (EtOH- treated cells).

### Oligonucleotide retrieval immunoprecipitation (ORiP)

The XPC- and XPA-DNA-interaction was tested using oligonucleotide retrieval immunoprecipitation (ORiP) as previously described^44^. HEMn were treated with melatonin or its metabolites for 24 h in complete melanocyte medium and 2% chFBS. Cells were further exposed to either 25 mJ/cm^2^ or 50 mJ/cm^2^ or non UVB-treated (0 mJ/cm^2^). After UVB treatment, cells were incubated in fresh medium supplemented with melatonin or its metabolites for an additional 1 or 3 h, before they were harvested by scraping and pelleted. Soluble nuclear extract was obtained as previously described^44^. Synthetic oligonucleotides (Molecular Beacons) were assembled to form 5′-biotinylated duplex DNA fragment that provide a substrate for NER proteins. A 30-nt oligonucleotide, 5′-CTCGTCAGCATCTTCATCATACAGTCAGTG-3′, was exposed to 10 J/m^2^ of UVC, annealed and ligated with two oligonucleotides as previously described^44^. After indicated treatments, soluble nuclear extracts (50 µg) were incubated with the biotinylated oligonucleotide in streptavidin-coated 96 well plates (Thermo-Scientific) (0.01 nM per well) for 10 minutes at 30 °C. Wells were washed with 40 mM Tris-HCl (pH 7.5) containing 0.01% BSA (wash buffer) followed by fixation in 4% paraformaldehyde. After three washes, 2 µg of either anti-XPA or anti-XPC was added for 1 h at room temperature. Detection was accomplished using an HRP-conjugated anti-rabbit secondary antibody (Abcam) for 1 h followed by the addition of 1-Step Ultra TMB ELISA Substrate (Pierce) to each well and absorbance measured at 400 nm.

### Nitric oxide (NO^−^) assays

Nitrite levels (NO^**−**^) levels were measured using Griess reagent (1% sulfanilamide-0.1% *N*-1-naphthyl-ethylenediamine dihydrochloride in 2.5% phosphoric acid) (Sigma, St. Louis, MO) as previously described^60^. Melanocytes were seeded onto 60 mm culture plates. Cells were treated with melatonin or its metabolites at the concentration of 5 × 10^−5^ M, or ethanol (vehicle, dilution 5:1,000) for 24 h. After incubation, cells in cold 1×PBS were irradiated with the UVB intensity of 0 or 25 mJ/cm^2^. After 30 min of incubation at 37 °C cells were harvested by scraping. Cell pellets were further centrifuged at 3000 g for 5 min, supernatant was collected and mixed with an equal amount of Griess reagent. The generation of NO was determined by measuring the absorbance at 540 nm in a spectrophotometer of purple azo compound formed from the reaction between nitrates formed in samples and Griess reagent. Data were presented as concentration of nitite (µmol) per protein amount (mg), and further as percentile of control (EtOH- treated cells).

### H_2_O_2_ assays

Hydrogen peroxide (H_2_O_2_) levels were measured using a luminescence method^61^. Melanocytes were treated with melatonin, its metabolites at the concentration of 5 × 10^−5^ M, or ethanol (vehicle, dilution 5: 1,000) for 24 h. After incubation, cells in 1×PBS were irradiated with the UVB intensity 0 or 25 mJ/cm^2^. The generation of H_2_O_2_ was determined by measuring the luminescence of luminol (Sigma, St. Louis, MO) by H_2_O_2_ that is released by melanocytes 30 min after UV exposure. Aliquots from each dish were mixed with lumiol and horse radish peroxidase (Sigma, St. Louis, MO) in respiratory buffer and luminescence was measured in a Turner Luminometer (TD20/20) (Promega, Madison, WI). The specificity of the reaction was determined by treating separate UV-irradiated cells with 300 units/mL of catalase (Sigma, St. Louis, MO), which degraded H_2_O_2_ to H_2_O and O_2_. Data were presented as concentration of H_2_O_2_ (pmol) per 0.1 million cells and further as percentile of control (EtOH treated cells).

### Glutathione (GSH) assay

Glutathione (GSH) levels were measured using a fluorometric method^60^. Melanocytes were treated with melatonin, or its metabolites at the concentration of 5 × 10^−5^ M, or ethanol (vehicle, dilution 5:1,000) for 24 h. After incubation, cells were irradiated with the UVB intensity of 0 or 25 mJ/cm^2^. After UV exposure cells were further incubated for 1 h at 37 °C. Cells were harvested with trypsinization and washed with 1×PBS-EDTA, pH8. After centrifugation, cell pellets were resuspended in ice cold 5% meta-phosphoric acid (MPA) (Sigma, St. Louis, MO), sonicated, and centrifuged at 12,000 rpm for 5 min. After collection of supernatant, aliquots were taken for protein determination. Supernatant was further mixed with 1×PBS-EDTA buffer and OPAME (o-phthaldialdehyde (Sigma, St. Louis, MO) in methanol (Fisher, Pittsburgh, PA) and borate buffer (potassium-tetraborate, Sigma, St Lois, MO). Mixtures were further aliquoted in 96-well plate, incubated for 15 min at RT and GSH levels were determined by measuring the fluorescent signal at 350 nm excitation and 420 nm emission, on SpectraMax M2e instrument (Molecular Devices, Sunnyvale, CA). Data were presented as percentile of control (EtOH- treated cells).

### Reverse transcription polymerase chain reaction (*RT-PCR*)

HEKn cells were cultured for three days in 60 mm petri dishes (MidSci, St. Louis, MO). Cells were treated with melatonin, or its metabolites at the concentration of 5 × 10^−5^ M, or ethanol (vehicle, dilution 5: 1,000) for 24 h before UVB exposure and for additional 3 h post UVB exposure. Cells were exposed to different intensity UVB including: 0, 25, 50 or 75 mJ/cm^2^. After UV exposure cells were further incubated for 1 h at 37 °C. RNA from cells was extracted using Absolutely RNA RT–PCR Miniprep kit (Stratagene, La Jolla, CA) as previously described^62^. Reverse transcription reaction was performed using High Capacity cDNA reverse transcription Kit (Applied Biosystems, Foster City, CA). The reaction was performed with LightCycler 480 Probes Master (Roche Applied Science, Indianapolis, IN). The sequence and source of primers are shown in Table 1 in supplementary file. Real-time PCR was performed using Kapa SYBR Fast qPCR Master Mix (Kapa Biosystems, Boston, MA) performing 35 cycles (95 °C for 15 seconds, 60 °C for 30 sec, 72 °C 10 sec) in triplicates. Data was collected with a Roche Light Cycler 480, and the amount of mRNA was normalized by comparative Ct method, using B-actin as a housekeeping gene.

### siRNA transfection

Melanocytes were transfected with 50 nM NRF2 or scrambled siRNA (Santa Cruz Inc, Santa Cruz, CA) using lipofectamine plus (Invitrogen, Eugene, Oregon). Twenty-four hours after transfection, cells were treated for an additional 48 h with 5 × 10^−5^ M melatonin. RNA was isolated and used for gene expression analysis and, also, whole cell extracts were prepared for protein analysis.

### Immunoprecipitation analysis

HEKn cells were lysed in RIPA buffer (Cell Signaling, Beverly, MA, USA) on ice for 1 h. After centrifugation (15, 339 g for 20 min at 4 °C), the lysates were incubated with 25 *μ*l of Protein A/G PLUS-Agarose beads (Santa Cruz Inc., Santa Cruz, CA) and 1 *μ*g of normal goat serum for 3 h with shaking at 4 °C, pelleted by centrifugation for 10 min at 4 °C, and incubated overnight at 4 °C on a rocking platform with 25 *μ*l of Protein A/G PLUS-Agarose beads and 2 *μ*g of anti MEL1A (sc13186), anti-MEL1A (sc13179), anti-MEL1B (sc13177), or anti-MEL1B (sc28453) antibodies (Santa Cruz Inc., Santa Cruz, CA). Immunoprecipitates were collected by centrifugation, washed four times with RIPA buffer, separated by SDS–PAGE, and subjected to immunoblotting, as described previously^62^. The primary antibodies used for immunoblotting were goat polyclonal antibodies against MEL1A or MEL1B. The secondary antibody used was HRP anti-goat IgG.

Experiments were repeated three times and analysed using Student’s t-test or anova (*) and appropriate post hoc test, using Microsoft Excel and Prism 4.00 (GraphPad Prism 4 Software) and representative graphs have been prepared. Statistically significant differences are denoted in the figures and corresponding figure legends.

## Electronic supplementary material

supplementary info
