# Supplementary material for: Melatonin and its metabolites protect human melanocytes against UVB-induced damage: Involvement of NRF2-mediated pathways
Source: Sci Rep. 2017 Apr 28;7:1274. doi: 10.1038/s41598-017-01305-2 (PMC5430855; doi:10.1038/s41598-017-01305-2)
Supplement: Supplementary file 1 — supplementary info [file 41598_2017_1305_MOESM1_ESM.pdf]

Melatonin and its metabolites protect human melanocytes against UVB-induced damage: Involvement of NRF2-mediated pathways

Zorica Janjetovic[1, 2*], Stuart G. Jarrett[3], Elizabeth F. Lee[2*], Cory Duprey[2*], Russel J. Reiter[4], Andrzej T. Slominski[1, 2*,5, 6]

[1]Department of Dermatology, University of Alabama at Birmingham, Birmingham, AL, USA

[2]Department of Pathology and Laboratory Medicine, University of Tennessee HSC, Memphis, TN, USA

[3]Department of Toxicology and Cancer Biology, The Markey Cancer Center, College of Medicine, University of Kentucky, Lexington, KY, USA

[4]Department of Cellular & Structural Biology, UT Health Science Center, San Antonio, TX, USA

[5]Comprehensive Cancer Center, Cancer Chemoprevention Program, and Nutrition Obesity Research Center, University of Alabama at Birmingham, Birmingham, AL, USA

[6]VA Medical Center, Birmingham, AL, USA

*Represents previous affiliation, where the project was started

Legend to Supplementary Figures

Supplementary Figure S1. Melatonin receptors are poorly expressed in human melanocytes. Nontreated HEMn cells were lysed in RIPA buffer and equal amounts of lysates were divided into four separate tubes and incubated with Protein A/G PLUS-Agarose beads and anti MEL1A (sc13186) (1), anti-MEL1A (sc13179) (2), anti-MEL1B (sc13177) (3), or anti-MEL1B (sc28453) (4) antibodies. Immunoprecipitates were separated by SDS–PAGE, immunoblotted with goat polyclonal antibodies against MEL1A (half of the membrane where lysates were incubated with MELA antibody) or MEL1B (the other half of the membrane where lysates were incubated with MELB antibody). The secondary antibody used was HRP anti-goat IgG.

Supplementary Figure S2. Melatonin and its metabolites induced the expression of antioxidative enzymes and NRF2 related genes. Human epidermal melanocytes were incubated with melatonin or its metabolites at the concentration of $5x10^{-5}$ M for 24 h before UV exposure. After cells were exposed to UVB, they were incubated again with fresh melatonin or its derivatives for 3 h. Cells were further harvested for RNA isolation. RT-PCR was performed using the equal amount of cDNA. B-actin served as internal control. Data were analyzed using t-test. The *p* value *, <0.05; **, <0.01; ***, <0.001, were determined *vs.* vehicle (EtOH, ethanol) control.

Supplementary Figure S3. Melanocytes used for all experiments were isolated from neonatal foreskin of African American donors. Cells in their third passage were used for the treatment with ethanol, melatonin, AFMK, 6-OHM, 5-MT or NAS 24 h before the exposure to UVB. Cells were further incubated for 3 h or 24 h in fresh media with compounds and harvested. Cell morphology did not change significantly when cells were exposed to UVB. Representative pictures of cells treated with vehicle (E) or melatonin (M) for additional 3 h or 24 h after UVB exposure (0, 25 and 50 mJ/cm^2) were taken under the light microscope magnification 10x (A). Melanin content remained similar among cells after treatment as seen as the dark color of cell pellets: UV exposure 0 mJ/cm^2 (B, D) or 50 mJ/cm^2 (C, E) of UVB, for 3 (B, C) or 24 h (D,

E). Densitometric analysis of the intensity of the cell pellets 24h after treatment was performed using Image J and presented in a graph (F).

Table 1: RT-PCR primers

Gene	Left primer	Right primer
Catalase CAT	CGTGCTGAATGAGGAACAGA	AGTCAGGGTGGACCTCAGTG
Glutathione peroxidase GPx	ACGATGTTGCCTGGAACTTT	GATGTCAGGCTCGATGTCAA
Superoxide dismutase Cu/Zn–SOD	GGCAAAGGTGGAAATGAAGA	GGGCCTCAGACTACATCCAA
Superoxide dismutase Mn–SOD	GCTCATGCTTGAGACCCAAT	CACCCGATCTCGACTGATTT
Heme oxygenase-1 (HO-1)	CCAGCGGGCCAGCAACAAAGTGC	AAGCCTTCAGTGCCCACGGTAAGG
Transcription factor NF-E2-related factor 2 (NRF2)	TTCTGTTGCTCAGGTAGCCCC	TCA GTTTGGCTTCTGGACTTGG
Glutamylcysteine synthetase (GCS)	TTGCAGGAAGGCATTGATCA	GCATCATCCAGGTGTATTTTCTCTT
Glutathione-S-transferase (GSTP1)	CCTGGTGGACATGGTGAATGA	CCTGGTGCAGATGCTCACATAGT
Glutathione reductase (GR)	GAGATGGCAGGGATCCTGTCAGC	GAGATGGCAGGGATCCTGTCAGC
NAD(P)H dehydrogenase, quinone2 (NQO2)	TTGACATCCCAGGATTCTACG	CCGTGGTTACGGAAAGGAG
Bata actin	CCAACCGCGAGAAGATGA	CCAGAGGCGTACAGGGATAG

The source of primers is described in following articles: CAT, GPx, Cu/Zn-SOD, and Mn-SOD[1] [2]; HO-1 [3]; NRF2 [4]; GCS [5]; GSTP1 [6]; GR [7]; NQO2 [8].

References:

1 Galano, A., Tan, D. X. & Reiter, R. J. On the free radical scavenging activities of melatonin's metabolites, AFMK and AMK. *J Pineal Res* **54**, 245-257, doi:10.1111/jpi.12010 (2013).

2 Fischer, T. W., Kleszczynski, K., Hardkop, L. H., Kruse, N. & Zillikens, D. Melatonin enhances antioxidative enzyme gene expression (CAT, GPx, SOD), prevents their UVR-induced depletion, and protects against the formation of DNA damage (8-hydroxy-2'-deoxyguanosine) in ex vivo human skin. *J Pineal Res* **54**, 303-312, doi:10.1111/jpi.12018 (2013).

3 Colombrita, C., Lombardo, G., Scapagnini, G. & Abraham, N. G. Heme oxygenase-1 expression levels are cell cycle dependent. *Biochem Biophys Res Commun* **308**, 1001-1008 (2003).

4 Zhang, T. *et al.* Luteolin modulates expression of drug-metabolizing enzymes through the AhR and Nrf2 pathways in hepatic cells. *Arch Biochem Biophys* **557**, 36-46, doi:10.1016/j.abb.2014.05.023 (2014).

5 Nguyen, T., Sherratt, P. J., Huang, H. C., Yang, C. S. & Pickett, C. B. Increased protein stability as a mechanism that enhances Nrf2-mediated transcriptional activation of the antioxidant response element. Degradation of Nrf2 by the 26 S proteasome. *J Biol Chem* **278**, 4536-4541, doi:10.1074/jbc.M207293200 (2003).

6 Brasch-Andersen, C. *et al.* Possible gene dosage effect of glutathione-S-transferases on atopic asthma: using real-time PCR for quantification of GSTM1 and GSTT1 gene copy numbers. *Hum Mutat* **24**, 208-214, doi:10.1002/humu.20074 (2004).

7 Kim, S. J. *et al.* Glutathione reductase plays an anti-apoptotic role against oxidative stress in human hepatoma cells. *Biochimie* **92**, 927-932, doi:10.1016/j.biochi.2010.03.007 (2010).

8 Slominski, A. *et al.* The role of melanogenesis in regulation of melanoma behavior: melanogenesis leads to stimulation of HIF-1alpha expression and HIF-dependent attendant pathways. *Arch Biochem Biophys* **563**, 79-93, doi:10.1016/j.abb.2014.06.030 (2014).

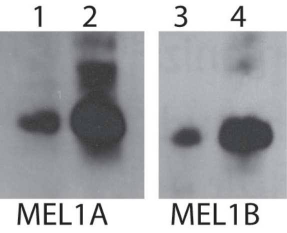
1
2
3
4
MEL1A
MEL1B

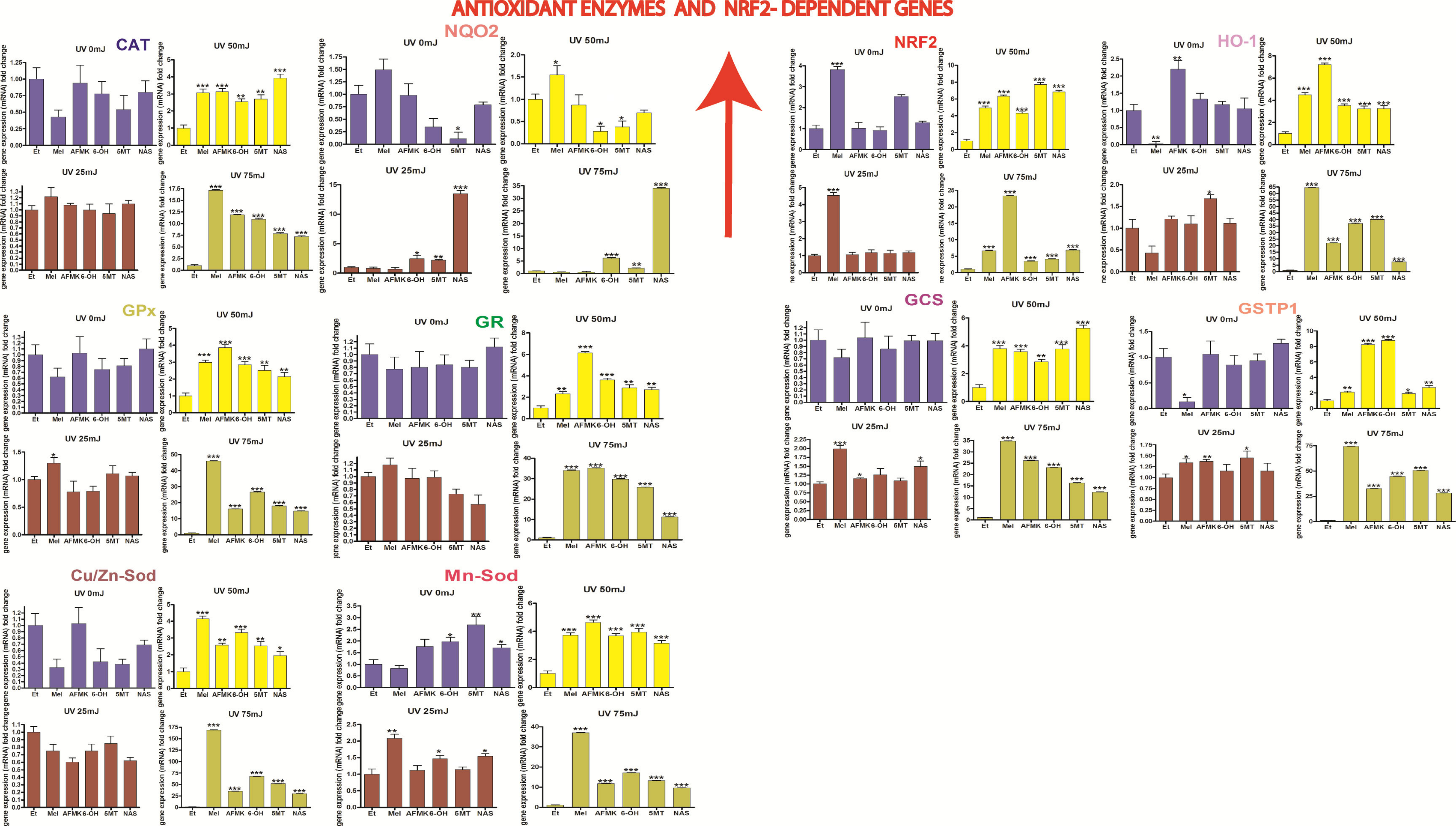
ANTIOXIDANT ENZYMES AND NRF2- DEPENDENT GENES
CAT
NQO2
NRF2
HO-1
GPx
GR
GCS
GSTP1
Cu/Zn-Sod
Mn-Sod
UV 0mJ
UV 25mJ
UV 50mJ
UV 75mJ
gene expression (mRNA) fold change
Et
Mel
AFMK
6-OH
5MT
NAS

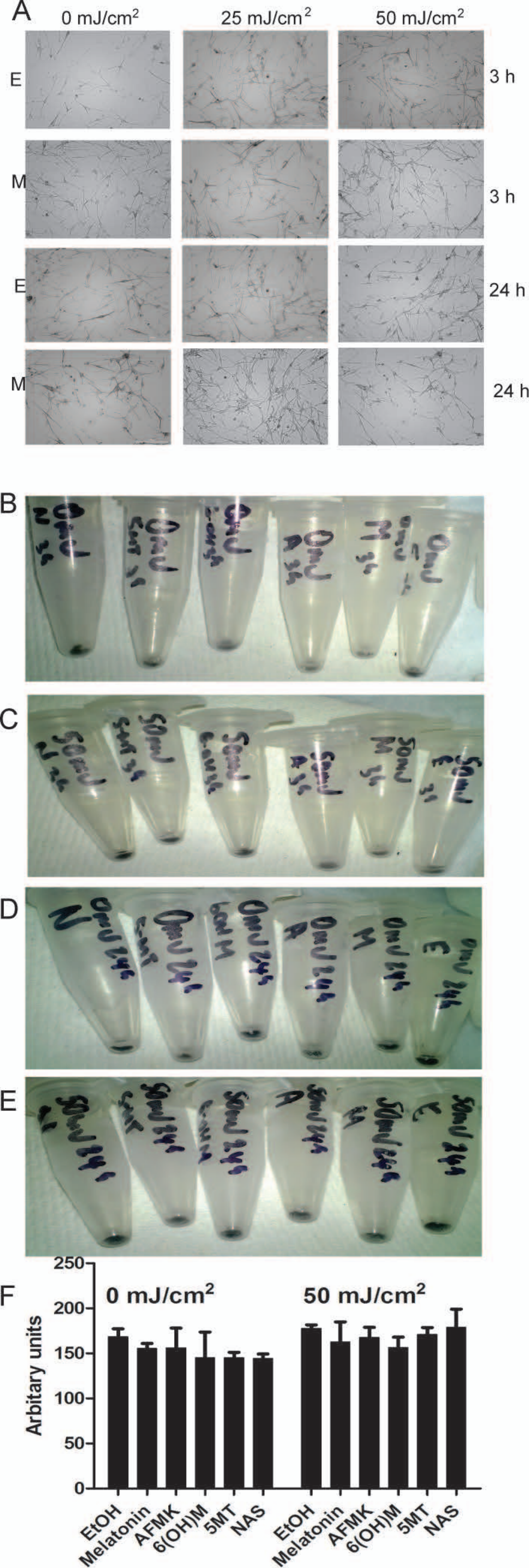
A
0 mJ/cm2
25 mJ/cm2
50 mJ/cm2
E
3 h
M
3 h
E
24 h
M
24 h
B
C
D
E
F
0 mJ/cm2
50 mJ/cm2
Arbitary units
250
200
150
100
50
0
EtOH
Melatonin
AFMK
6(OH)M
5MT
NAS
EtOH
Melatonin
AFMK
6(OH)M
5MT
NAS
